# Comparative Analysis of Robotic-Assisted Versus Laparoscopic Appendectomy: A Review

**DOI:** 10.7759/cureus.63488

**Published:** 2024-06-29

**Authors:** Srinivasa Reddy, Darshana Tote, Anup Zade, Kesav Sudabattula, Tushar Dahmiwal, Akansha Hatewar, Dushyant Bawiskar

**Affiliations:** 1 General Surgery, Jawaharlal Nehru Medical College, Datta Meghe Institute of Higher Education and Research, Wardha, IND; 2 Sports Medicine, Abhinav Bindra Targeting Performance, Bangalore, IND

**Keywords:** open surgical repair, appendicitis, laparoscopic appendectomy, robotic-assisted surgery, appendectomy

## Abstract

Appendectomy ranks among the most common surgical procedures. Laparoscopic appendectomy has become increasingly popular among certain surgeons. Even laparoscopic appendectomy is considered the gold standard; many surgical subspecialties have adopted robotic surgery in the past 10 years. The robotic system is recognized for enhancing stability, visualization, precision, and spatial flexibility. Surgeons can operate with enhanced dexterity, reduced tremors, three-dimensional visualization, up to 10 times magnification, and control over four arms thanks to improved ergonomics that allow them to sit at a customizable console. The purpose of this study is to evaluate and compare the overall effects, such as intraoperative time, postoperative recovery, feasibility for surgeons, and cost-effectiveness, of robotic-assisted appendectomy and laparoscopic appendectomy through the available literature. It was found that both robotic and laparoscopic surgeries work well for appendectomy, but in some studies, it was found that robotic surgery comes with the perks of shorter hospital stays and quicker recovery, even though it is more expensive, and in some studies, no differences were observed in patient recovery postoperatively. Laparoscopic surgery is still a highly effective and commonly used method, with proven advantages over open appendectomy, despite taking longer for the procedure. We need more studies to fully understand the advantages and disadvantages of robotic surgery, especially when it comes to cost-effectiveness and wider health outcomes.

## Introduction and background

With a lifetime incidence of 7%-9%, acute appendicitis is a common cause of acute surgical abdominal pain. Consequently, appendectomy ranks among the most common surgical procedures [1]. The first description of the open approach to appendectomy came from McBurney [2]. Because of its good efficacy and safety, it has evolved into the preferred standard treatment for acute appendicitis and has remained mostly unchanged for the past 100 years. Subsequently, when Semm performed the first laparoscopic appendectomy (LA) in 1983, the procedure gradually gained popularity. Nonetheless, there is still debate in the literature regarding the best way to remove the inflamed appendix. LA has become increasingly popular among certain surgeons, while some surgeons are still doubtful that it will replace the more straightforward open appendectomy (OA) [3]. Nevertheless, LA is considered the gold standard for treating appendicitis owing to its advantages over OA, including a lower risk of wound infections, less pain after surgery, and a shorter hospital stay [4]. There have been a few prospective randomized controlled trials comparing LA and OA, taking into account research that has been published in English. While some research found that LA was superior to OA in terms of wound healing, more rapid recovery, and earlier diet resumption, other research found none of these advantages, and some even supported traditional appendectomy [5,6].

The field of minimally invasive surgery (MIS) has advanced significantly in more than a century since gynaecologist Dimitri Ott used a head mirror and a speculum to examine a woman's peritoneal cavity through a culdoscopic opening in 1901 [7,8]. In 1985, nearly 85 years later, Erich Mühe carried out Germany's first laparoscopic cholecystectomy [9]. The creation of remote robotic telesurgery, which was initially used in combat and is now used in many surgical specialties, marked a further advancement in medical technology [10]. It has been demonstrated over time that this development results in, at the very least, technical outcomes that are on par with or even better than those obtained from similar laparoscopic procedures [11-13]. The robotic platform presents a significant difference in the operative experience for surgeons. Surgeons can operate with enhanced dexterity, reduced tremors, 3D visualization, up to 10 times magnification, and control over four arms thanks to improved ergonomics that allow them to sit at a customizable console. These features make MIS easier for non-laparoscopy operators to understand [14,15].

Almost all surgical subspecialties have adopted robotic surgery as a routine procedure in the past 10 years, as its use has increased in several surgical specialties [16,17]. As surgeons gain proficiency with the robot, they can employ fewer instruments in more creative ways, which reduces the need for instrument exchanges and lowers operating room time and expense [18]. In laparoscopic procedures, the robotic system is recognized for enhancing stability, visualization, precision, and spatial flexibility [19].

It has been demonstrated that minimally invasive procedures are not only more patient-friendly in terms of a shorter recovery period and less pain but also the approach that patients prefer, leading to increased satisfaction [20]. It has been demonstrated to lower the possibility of transmission of communicable diseases to surgeons and other staff by enabling them to work remotely from both the patient and one another [21,22]. The main purpose of this study is to evaluate and compare the effects of robotic-assisted appendectomy and LA through the available literature. This study attempts to determine the benefits, drawbacks, and possible ramifications of each surgical technique in the treatment of appendicitis by reviewing the contents of existing literature.

## Review

Methodology

**Figure 1:**
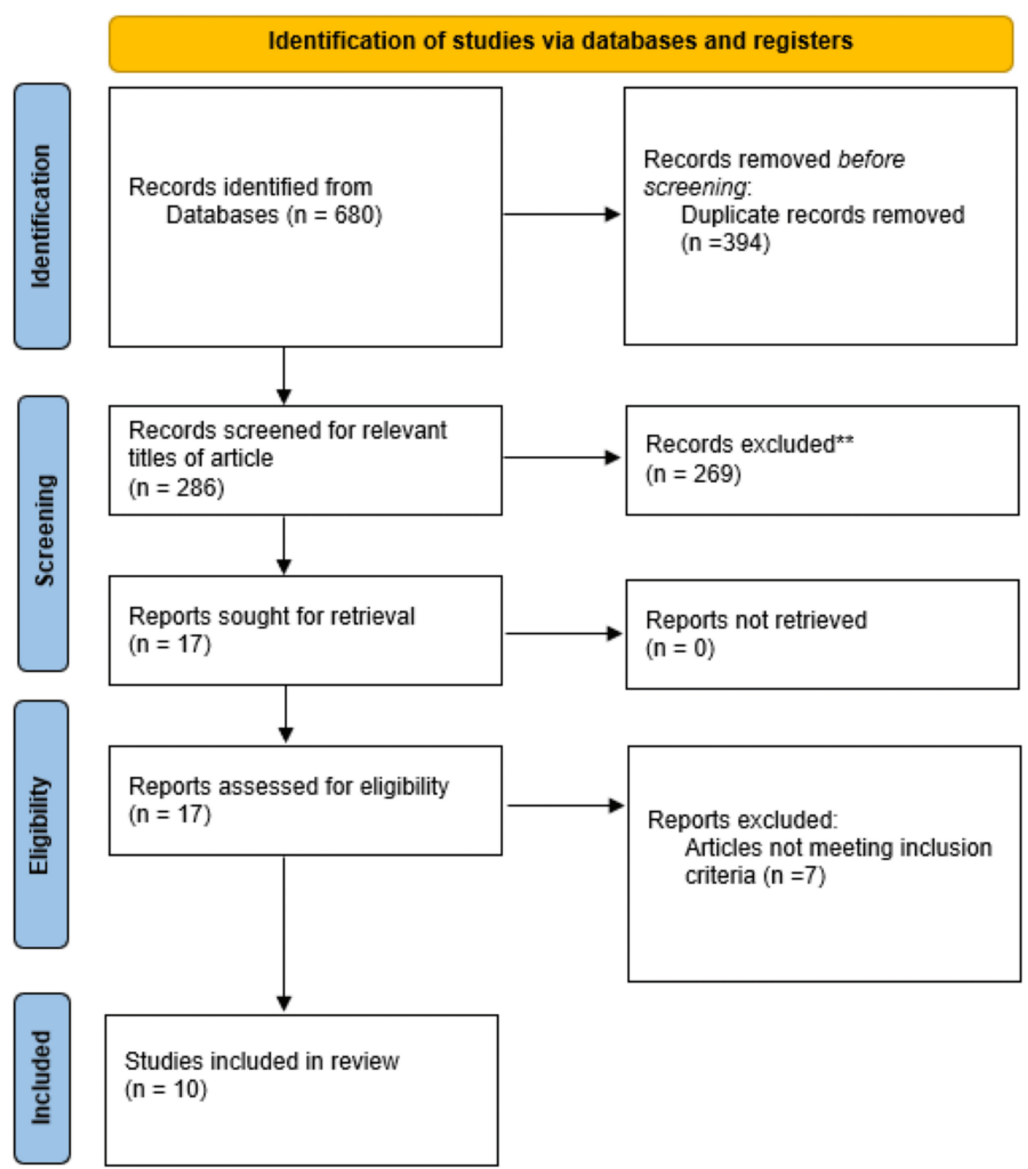
PRISMA flowchart PRISMA: Preferred Reporting Items for Systematic Reviews and Meta-Analyses

A search was conducted for articles published between September 1995 and October 2023 on electronic databases (PubMed, Google Scholar) using the search terms "Robotic-assisted appendectomy" and "Laparoscopic appendectomy" in the abstract or title. During the search, the study design criteria, publication type, and language limitations were applied. The inclusion criteria for the study included non-randomized controlled trials (non-RCTs), RCTs, and studies that looked at the effects of laparoscopic and robotic-assisted appendectomy published in English in peer-reviewed journals. Studies unrelated to the investigation or published in non-peer-reviewed journals were eliminated. RCTs, experimental studies, literature reviews, and other study designs were all utilized. Following a preliminary investigation, 680 articles were found in the search database; we then eliminated 394 articles that were duplicates. A total of 269 studies were excluded due to their irrelevance to the topic. After reviewing the full text of 17 articles, we excluded seven studies due to their failure to meet the inclusion criteria. Finally, 10 articles were included in the final review. Figure 1 shows a summary of the selected publications based on the Preferred Reporting Items for Systematic Reviews and Meta-Analyses (PRISMA) guidelines.

Robotic-assisted appendectomy

Surgical Outcomes and Recovery

There have been studies evaluating RA and highlighting its advantages and disadvantages. According to Becker et al., RA takes longer surgical time compared to LA, but the postoperative stay was shorter for the patients treated with RA. Rifai et al. also found that recovery time after RA was noticeably shorter, even though the actual surgical time for both laparoscopic and robotic methods was the same. This implies that RA may have a positive effect on recovery time [23,24].

Safety and Efficacy

Akl et al. performed RA appendectomy as a combined procedure with other gynaecological conditions. The authors concluded that RA could be done safely and effectively without interrupting the total time of other gynaecological procedures and without the requirement to switch to regular laparoscopy. Therefore, it appears that RA can be smoothly integrated into more complex surgeries without compromising safety. According to Yi et al., using the "Micro Hand S" robot system for RA was also beneficial. They did not encounter any technical issues or complications during surgery, and the patients were recovering well at a three-month follow-up [25,26].

Cost Considerations

A study by Quilici et al. took a close look at the costs associated with RA surgeries. Their findings revealed that the average direct and total costs per case for RA appendectomies were significantly higher than those for LA. Compared to LAs, which cost $3081 and $7709, the few robotically assisted appendectomies had an average direct cost per case of $7894 and an average total cost per case of $13,210. They pointed out that the increased costs may not necessarily lead to better clinical care outcomes for gastrointestinal surgeries [27].

Specific Case Studies

Another study by Kelkar et al. showcased the successful completion of emergency robotic-assisted surgeries in four cases, with minimal blood loss during the surgery and reasonable surgical durations ranging from 80 to 135 minutes. Additionally, Orcutt et al. discussed the use of robotic-assisted surgery for treating patients with appendiceal mucoceles. They suggested that it provides safety and effectiveness comparable to traditional laparoscopy, along with added benefits for specific conditions [28,29]. The study by Becker et al. revealed that RA witnessed a shorter postoperative stay (0.7 days vs. 1.3 days, p < 0.01) but a longer median operation time (71.0 minutes vs. 46.0 minutes, p < 0.01). Overall, both robotic and laparoscopic surgeries work well for appendectomy, but robotic surgery comes with the benefits of shorter hospital stays and quicker recovery, even though it is more expensive. Laparoscopic surgery is still a highly effective and commonly used method, with proven advantages over OA, despite taking longer time for the procedure. We need more studies to fully understand the advantages and disadvantages of robotic surgery, especially regarding cost-effectiveness and broader health outcomes.

Laparoscopic appendectomy

Many studies have evaluated the effects of LA compared to OA and other methods. According to Biondi et al., LA comes with some clear advantages over OA, such as shorter hospital stays, less need for analgesics after surgery, quicker return to daily living activities, and a lower risk of wound infection. However, it is worth noting that these benefits do come with slightly higher costs. In a study by Khalil et al., it was found that although LA resulted in less pain after surgery, the procedure itself took longer compared to OA in LA, the surgery took 48.26 ± 12.82 minutes, while in OA, it took 31.36 ± 11.43 minutes (p < 0.001). They concluded that while the main results for LA and OA were similar, the advantages of less pain following surgery were somewhat offset by the longer surgical recovery time for LA. Similarly, Martin et al. found that there were not any significant differences in the time it took to go back to work or normal activities between LA and OA, even though LA did take longer to perform [30-32]. Table 1 includes a summary of the studies included in the review.

**Table 1:** Summary of articles reviewed to compare robotic-assisted appendectomy and laparoscopic appendectomy LA: laparoscopic appendectomy; OA: open appendectomy; RA: robotic-assisted appendectomy

Author name and year	Patient population	Study group	Treatment	Author’s perspective
Timothy Becker et al. (2023) [23]	Patients undergoing appendectomy	49,850	LA was done for 49,800 patients, and RA appendectomy for 50 patients.	There was no difference in co-morbidities between the groups. RA requires a longer mean surgery time but a shorter postoperative stay for the patient. As there was a smaller population for RA, the author recommended further studies with larger patient populations.
Ahmad Oussama Rifai et al. (2023) [24]	Patients with acute appendicitis or acute cholecystitis if they underwent an appendectomy or cholecystectomy	461	191 appendectomies and 270 cholecystectomies were performed on the 461 study participants. 110 laparoscopic and 81 robotic appendicectomies were performed on the patients who received appendectomies. Out of the patients who received cholecystectomies, 165 had a robotic cholecystectomy, and 105 had a laparoscopic cholecystectomy.	For the appendectomies and cholecystectomies, the surgical intraoperative times for laparoscopic and robotic-assisted techniques were equal; however, the robotic approach's post-operative period and recovery were noticeably shorter. In this study, Surgeon B—a robotic surgeon with full training—performed all robotically assisted surgeries. Although it's unclear if robotic training enhances laparoscopic skills, they assumed that surgeons with the same skill level would have similar laparoscopic skills. A robotic approach may lower the chance of switching to open procedures and is a more adaptable method for managing technically challenging cases.
Mohamed N. Akl et al. (2008) [25]	Patients undergoing appendectomy who also had gynecological issues	107	A robotic appendectomy was performed in conjunction with other robotic gynecological procedures on 107 patients in total. Seven patients with ovarian cancer and ten patients with endometrial cancer comprised the 17 patients (15%) who underwent the procedure in addition to robotic staging of their cancers. Ninety (85%) patients underwent a prophylactic appendectomy in conjunction with other gynecological procedures for benign disease; 62 (58%) underwent hysterectomy with or without adnexectomy; 23 (21%) underwent endometriosis excision; 11 (10%) underwent adnexectomy; 9 (8%) underwent presacral neurectomy; 5 (4%) underwent myomectomy; and 2 (1.8%) underwent upper vaginectomy.	Appendiceal pathological abnormalities are more common in gynecological patients with pelvic pain and ovarian cancer. Robotic appendectomy should be considered in patients undergoing robotic pelvic surgery for pelvic pain or ovarian cancer. The technique for robotic appendectomy can be performed safely and effectively without significantly altering the total time of the concomitant procedure and without the need for conversion to conventional laparoscopy.
Bo Yi et al. (2016) [26]	Patient with acute appendicitis	2	Robotic appendectomy was studied with 2 pts on one arm, and robotic perforation repair was performed on one patient with a gastric perforation. During these procedures, the "Micro Hand S" robot system was utilized initially. Every patient was monitored for three months, during which the duration of the robotic procedure, intraoperative blood loss, pre- and postoperative variations in standard blood tests, liver and renal function tests, and significant complications were noted.	The benefits of low cost and ease of use are provided by the first surgical robot system made in China and found to be safe and practicable for use in a limited number of patients. Both technical issues and intraoperative complications were absent. Patients showed no signs of adverse reactions at a three-month follow-up, and they were found to be progressing well. To assess its effects in diverse scenarios, more research needs to be done.
Sonia T. Orcutt et al. (2017) [29]	Patients with appendiceal mucoceles	2	A robotic approach and a hand-assisted laparoscopic approach were the two minimally invasive methods of appendectomy that were successfully used to treat these patients.	Due to the risk of perforation, laparoscopy has historically been ruled out for appendectomy for mucoceles. However, two new minimally invasive methods have been introduced: robotic-assisted laparoscopy and hand-assisted laparoscopy. These methods offer advantages comparable to laparoscopy, but they may be safer and enable patients with appendiceal mucoceles to receive successful surgical treatment.
Dhananjay Kelkar et al. (2021) [28]	Patients with acute appendicitis	Altogether, 30 patients (emergency RA: 4)	There were 9 cholecystectomies, 6 robot-assisted total laparoscopic hysterectomies, 4 appendectomies, 5 cases of diagnostic laparoscopy, two oophorectomies, 2 procedures for fallopian tube recanalization, an ovarian cystectomy, and a salpingo-oophorectomy.	In all four patients, RA was completed successfully. The estimated intraoperative blood loss was minimal, and the surgical procedure took anywhere from 80 to 135 minutes.
Philippe J. Quilici et al. (2021) [27]	Patients referred for various abdominal surgeries, including acute appendicitis	34,984 (unspecified number for RA)	In comparison to laparoscopic appendicectomies, which cost $3081 and 7709, the few robotically assisted appendectomies had an average direct cost per case of $7894 and an average total cost per case of $13,210.	In certain surgical specialties, robotic technology improves the skills of our surgical providers and could make the higher costs of these procedures justified. However, the results of this study are sufficient to conclude that certain intra-abdominal procedures and robotic technology for GI do not seem to improve clinical care despite being significantly more costly than alternative surgical methods that yield equivalent clinical results.
Jawad Khalil et al. (2011) [31]	Patients with acute appendicitis	160	Patients were equally divided into 2 groups. Patients in group A were subjected to LA and for group B OA. Age, gender, and key outcome measures (hospital stay, length of surgery, and postoperative complication) were among the information that was collected and examined.	The LA group experienced noticeably less postoperative pain. In LA, however, the length of the operation was greater. Nevertheless, no variation was observed in the remaining primary outcome measures. The authors arrived at the conclusion that, in terms of primary outcome measures, LA is not superior to OA and is equal to it. This is because the longer surgical duration offsets any benefit from reduced postoperative pain.
L C Martin et al. (1995) [32]	Patients with acute appendicitis	169	81 patients were randomized for laparoscopic appendectomy and 88 for open repair. Out of 81, 13 were converted to open repair.	Between the laparoscopic and open groups, there was no statistically significant difference in return to work or activity. In the laparoscopic group, the procedure took noticeably longer. In terms of complications, length of stay in the hospital, expense, and time to resume activities and work, laparoscopic and open appendectomy are similar. The laparoscopic technique required a longer amount of time during the procedure. For the typical appendicitis patient, laparoscopic appendectomy does not provide a discernible advantage over the open procedure.
Antonio Biondi et al. (2016) [30]	Patients with acute appendicitis	593	283 patients underwent laparoscopic appendectomy, and conventional appendectomy was done for 310 patients.	The laparoscopic approach to appendectomy is a safe and effective surgical technique that comes with only slightly higher hospital costs. Compared to the open method, the laparoscopic approach offers clinically beneficial advantages such as a shorter hospital stay, less need for postoperative analgesia, early nutrition acceptance, an earlier return to work, and a lower rate of wound infection.

Discussion

The comparative analysis of RA and LA has revealed notable distinctions in the effectiveness of each approach, recovery period, and financial impact. First, RA implies several one-of-a-kind postoperative benefits since multiple sources point to a shorter period of hospitalization and time of recovery in comparison to LA surgeries. While this factor may improve patients’ postoperative results and diminish the extent of follow-up care, on the one hand, on the other, RA surgeries take longer duration and cause significantly higher expenditures. This factor calls the appropriateness of RA treatment into question since it does not consistently deliver better clinical results than LA.

LA, on the other hand, has been proven to be a reliable and efficient form of surgery. Although its operative times are longer than open repair but still shorter than RA, it leads to short hospital stays, decreased postoperative need for analgesics, and an earlier return to normal despite slightly rising direct costs. The increasing preference for this method of appendectomy has implied these benefits over OA. Robotic surgery has many benefits, but it also has many disadvantages, such as limited availability and the requirement for additional specialized surgical robotic training. Furthermore, one of the primary drawbacks of robotic surgery is still its high cost in comparison to laparoscopic or open surgery. Specialized training is necessary for robotic surgery, and compared to other surgical techniques, the cost of purchasing, using, and maintaining a surgical robotic system is much higher [27]. LA may remain a reliable, cost-effective, and clinically beneficial option [33,34]. The limitation of this review is the number of studies selected for RA and the availability of randomized trials for RA for appendectomy. Still, many available studies were included, but future studies are needed to further evaluate the effect of robotic-assisted surgeries in such cases.

## Conclusions

Both RA and LA have their advantages and disadvantages in managing appendicitis. RA offers a shorter postoperative recovery but takes longer to perform and is costly. LA is a well-established, cost-effective procedure that is successfully used by all surgeons. RA is suitable for acute, complex cases, but more studies are needed to compare procedural times and address costs. Further research is necessary to improve the cost-effectiveness of RA and determine its role in appendectomies.
